# Dedifferentiated Low-Grade Central Osteosarcoma of the Mandible

**DOI:** 10.1155/2022/9321728

**Published:** 2022-01-21

**Authors:** Kenji Yamagata, Naomi Ishibashi-Kanno, Ryota Matsuoka, Fumihiko Uchida, Satoshi Fukuzawa, Hiroki Bukawa

**Affiliations:** ^1^Department of Oral and Maxillofacial Surgery, Institute of Clinical Medicine, Faculty of Medicine, University of Tsukuba, Japan; ^2^Department of Diagnostic Pathology, Institute of Clinical Medicine, Faculty of Medicine, University of Tsukuba, Japan

## Abstract

We present the first, to our knowledge, case of a dedifferentiated low-grade central osteosarcoma (LCOS) of the mandible. A 48-year-old Japanese woman underwent enucleation under general anesthesia after a diagnosis of ossifying fibroma. At the second recurrence, the pathological diagnosis after biopsy was of sarcoma with MDM2(+) and CDK4(+) immunohistochemical staining results. Hemimandibulotomy, supraomohyoid neck dissection, and free-flap reconstruction with a rectus abdominal flap were performed. A retrospective reevaluation of the first specimen with additional immunohistochemical staining for MDM2 and CDK4 yielded a final diagnosis of dedifferentiated LCOS. The patient showed no recurrence or lung metastasis 3 years after the final surgery.

## 1. Introduction

Low-grade osteosarcoma (LGOS) is rare and includes low-grade central OS (LCOS) and parosteal OS. LCOS represents less than 2% of all OSs reported in the literature [1]. In general, OS of the jaw is a high-grade lesion and relatively rare [2, 3]. The biological behavior of LGOS is more indolent than that of high-grade conventional OS. Microscopically, LGOS is often misdiagnosed as a benign fibrous lesion and is most often confused with fibrous dysplasia. It has been reported that all benign fibrous and fibroosseous lesions and control-group tumors show negative results for *murine double-minute type 2* (MDM2) and/or *cyclin-dependent kinase 4* (CDK4), which are also markers for distinguishing between dedifferentiated LGOS and conventional high-grade OS [4]. Although one case of dedifferentiated parosteal OS of the maxilla has been reported in the literature on the oral and maxillofacial area [5], to our knowledge, LCOS has not been reported to date. We present the first case of this entity, which was retrospectively diagnosed as dedifferentiated LCOS of the mandible based on MDM2 and/or CDK4 immunohistochemical findings.

## 2. Case Presentation

**Figure 1:**
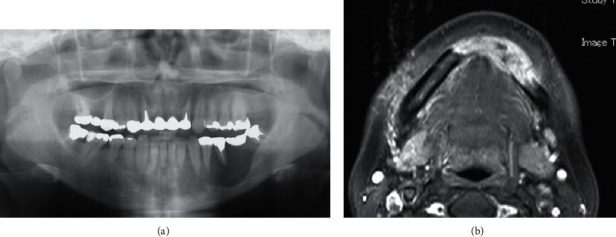
(a) Panoramic radiograph revealing a posterior well-demarcated radiolucent area and anterior irregular area. (b) Magnetic resonance images (T1 weighted) showing a tumor in the midline to the left side of the mandible with a high-signal mass measuring 42 × 13 mm.

**Figure 2:**
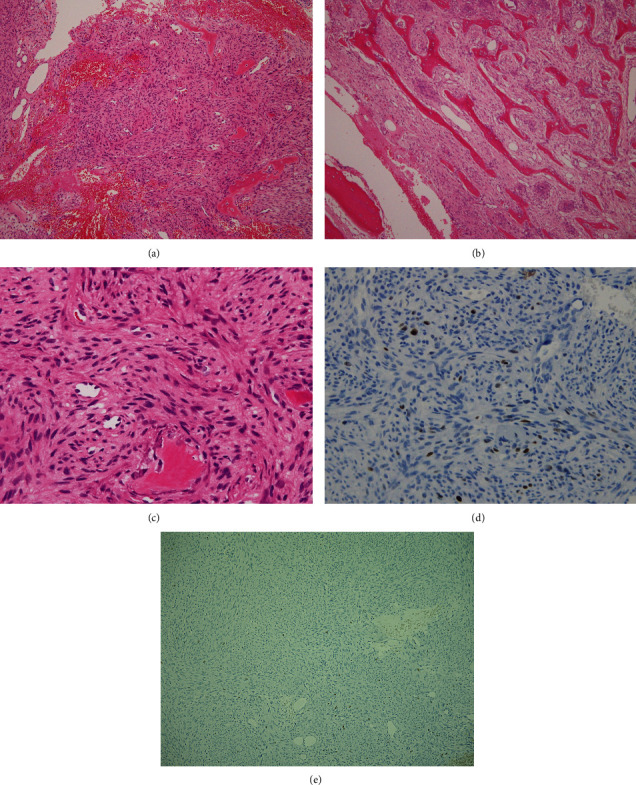
(a) Hematoxylin and eosin staining of the first enucleation (×100). (b) Hematoxylin and eosin staining after the first enucleation (×100). (c) Hematoxylin and eosin staining after the first enucleation (×200). The bundle of spindle cells runs across, and a storiform pattern is observed. Randomly formed woven bone is also observed. There is no atypia, and few mitoses are observed (2–3/50 high-power fields). (d) Immunohistochemical staining. The tumor is positive for MDM2(+). (e) Immunohistochemical staining: Ki-67 index of 2–3%.

A 48-year-old Japanese woman who showed radiolucency of the left mandible on panoramic radiography was referred to the Department of Oral and Maxillofacial Surgery, University of Tsukuba Hospital. Her medical history included hypertension and an ovarian cyst. Her face was symmetrical, and slight paresthesia of the lower lip and mentalis was noted. Diffuse elastic hard swelling was observed from the left canine to the second molar. Panoramic radiographs revealed a posterior well-demarcated radiolucent area and an anterior irregular area (Figure 1(a)). A well-enhanced high-signal area measuring 42 × 13 mm from the center to the left molar of the mandible was observed on T1-weighted magnetic resonance imaging (MRI) (Figure 1(b)). Biopsy was performed, and ossifying fibroma (OF) was diagnosed. The patient underwent enucleation under general anesthesia for OF. The pathological findings included a bundle of spindle cells running across each and storiform patterns. Randomly formed woven bone was observed. There was no atypia, and few mitoses were observed (2–3/50 high-power fields; HPFs). Retrospective immunohistochemical staining revealed MDM2(+), CDK4(-), and a Ki-67 index of 2–3% (Figure 2).

Primary recurrence occurred 1 year and 3 months after the first enucleation. A soft tissue mass-like granuloma appeared in the left molar area intraorally. The biopsy indicated granulation tissue with spindle cells. Panoramic radiography revealed an increase in the radiolucent area to the right side of the mandible. Bilateral mandibulectomy and reconstructive surgery with fibula were performed, and the spindle cell-like fibroblast tumor cells were found to be pathologically complicated and thick, forming irregular eosinophilic osteoid. The tumor had destroyed the cortex of the mandible, and the surgical margin was free. The nuclei were swollen and mildly oval. The atypia was mild, and 3 mitoses/50 HPFs were observed. The retrospective immunohistochemical staining findings were MDM2(+), CDK 4(-), and a Ki-67 index of 15%.

**Figure 3:**
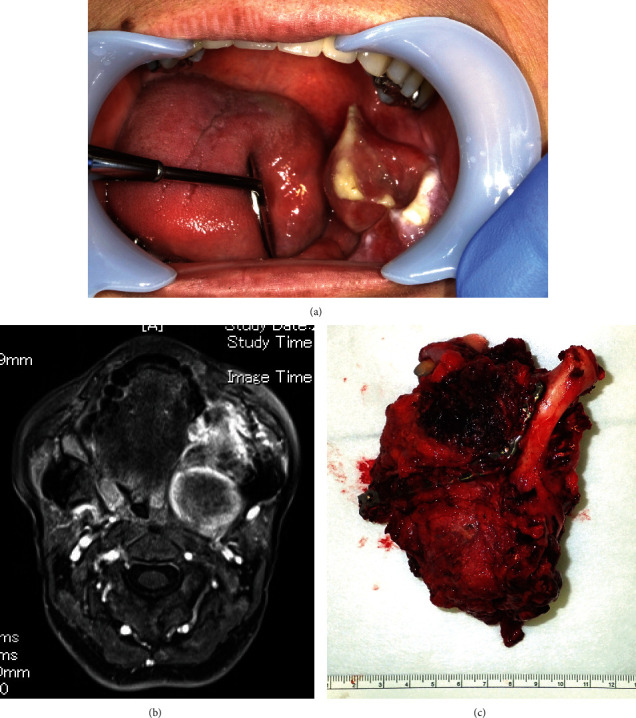
(a) Oral findings for the recurrent tumor. A growing mass is observed at the left posterior part of the mandible showing rapid growth approximately 6 years after the first enucleation. (b) Magnetic resonance image (short-term inversion recovery) depicting a 39 × 64 × 59 mm highly enhanced mass expanding from the left mandible to the parapharyngeal space. (c) The resected specimen.

**Figure 4:**
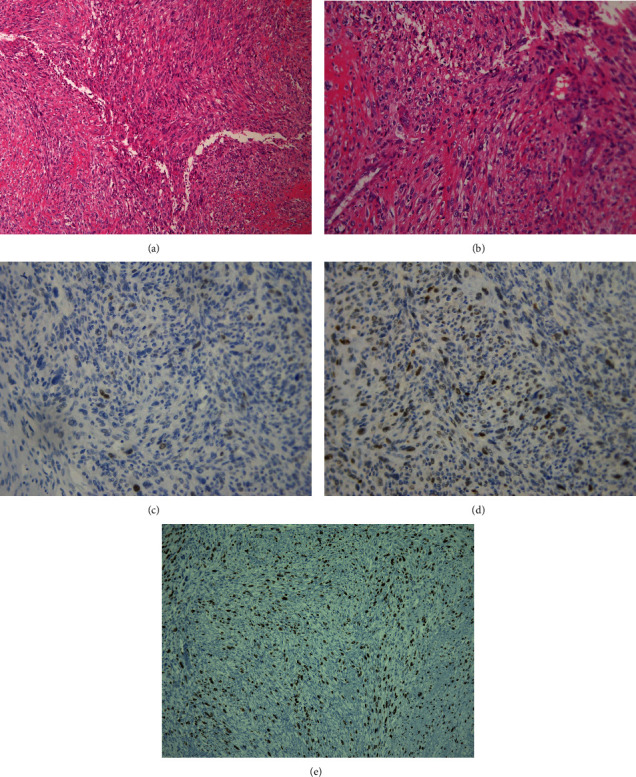
(a) Hematoxylin and eosin staining (×100). (b) Hematoxylin and eosin staining (×200). The spindle-like tumor cells appear to have formed thick fascicles with severe atypia around the tumor borders. The cells show more than 100 mitoses/50 high-power fields. There is no osteoid or necrotic tissue. (c) Immunohistochemical staining: positive results for CDK4. (d) Immunohistochemical staining: positive results for MDM2. (e) Immunohistochemical staining: Ki-67 index of 40%–50%.

Four years and 8 months after reconstructive surgery, a rapidly growing mass was observed in the left posterior part of the mandible (Figure 3(a)). A 39 × 64 × 59 mm highly enhanced mass expanding from the left mandible to the parapharyngeal space was observed on short-term inversion recovery MRI (Figure 3(b)). The pathological diagnosis after biopsy was of sarcoma with MDM2(+) and CDK4(+) immunohistochemical staining results. Hemimandibulotomy of the reconstructed mandible and supraomohyoid neck dissection with a titanium plate and free flap reconstruction with a rectus abdominal flap were performed under general anesthesia (Figure 3(c)). Pathologically, the spindle-like tumor cells were arranged in thick fascicles with severe atypia around the tumor borders and more than 100 mitoses/50 HPFs. Severe hyalinization was also observed. There was no osteoid or necrotic tissue. The results of immunohistochemical staining were as follows: *α*-SMA(+), desmin(-), h-caldesmon(-), pankeratin(-), S-100(+: sporadic), CD34(-), *β*-catenin(-), MDM2(+), CDK4(+), and Ki-67 (40%–50%) (Figure 4).

Retrospective reevaluation of the previous specimen with additional immunohistochemical staining for MDM2, CDK4, and Ki-67 was performed, and a final diagnosis of dedifferentiated LCOS (pT1N0, stage IIA: UICC 8^th^) was made. Postoperative chemotherapy and radiotherapy were not performed because of patient refusal. There was no recurrence or lung metastasis, and the patient's status has remained uneventful for 3 years after the final surgery.

## 3. Discussion

LGOS is rare and subdivided into parosteal OS and LCOS. Parosteal OS comprises a surface tumor representing 4%–5% of all OSs; it usually develops in the posterior surface of the distal femur in the third decade and in 15% to 43% of cases dedifferentiates into high-grade OS [6]. Only one report has described a dedifferentiated parosteal OS in the oral and maxillofacial region [5]. Conversely, LCOS accounts for 1%–2% of all OSs in the second to third decades and is mainly seen in the metaphysis of the long bones, e.g., the attachment of the tibia and femur. Involvement of the jawbone and axial and small tubular bones is rare. Very rarely, dedifferentiation can occur, resulting in conventional high-grade OS [6, 7]. To the best of our knowledge, previous reports describing dedifferentiated LCOS only involved the fibula and iliac region [8, 9]; our case was the first of dedifferentiated LCOS of the jaw.

Triantafillidou et al. [10] previously reported the clinical characteristics of OF, which is a fibroosseous tumor affecting the jaw and is composed of proliferating fibroblasts and osseous products that include bone and cementum-like material. OF is a slow-growing tumor of the jaw, and small or well-demarcated early tumors are treated with curettage or enucleation. In contrast, aggressive tumors that show rapid enlargement are treated with radical resection [10]. Wagner et al. reported that among all fibrous-osseous lesions, only fibrous dysplasia seems to be associated with a considerably increased risk of malignant transformation [11]. Our case was initially diagnosed as OF, distinguished from fibrous dysplasia, because the tumor was clinically well circumscribed and could be partially separated from normal bone during surgery. However, diagnosis in the absence of MDM2 and CDK4 immunohistochemical staining results may cause difficulty in distinguishing these tumors from fibrous-osseous lesions. The first enucleated specimen revealed MDM2(+) and CDK4(-) findings, and the decalcification may have weakened the immunohistochemical staining detection ability. Finally, our case was diagnosed as LCOS based on the clinical course, pathological morphology, and MDM2 and CDK4 immunohistochemical staining findings.

LCOS is also more likely to be misdiagnosed and inappropriately treated with an intralesional procedure because of overlap of its pathological characteristics with those of benign bone tumors [12]. Our patient was initially diagnosed with OF and underwent a resection procedure for benign tumors. However, 4 years and 8 months later, a clinically malignant tumor appeared, and OS was diagnosed following biopsy. Pathologically, during the three surgeries, Ki-67 changed from 2–3% to 15% and then to 40–50%. A previous study reported that all benign fibrous and fibroosseous lesions are negative for MDM2 and CDK4 [4]. In our case, MDM2 changed from a minor component to a positive one at the first enucleation. Based on these results, the initial diagnosis of OF was retrospectively corrected to a diagnosis of LCOS, and the tumor was finally diagnosed as dedifferentiated LCOS.

Dujardin et al. reported that MDM2 and/or CDK4 immunoreactivity was present in 89% of LGOS specimens. Although dedifferentiated LGOS and conventional high-grade OS involve different oncogeneses, they share histological features. Therefore, dedifferentiated LGOS is histologically indistinguishable from conventional OS. MDM2 and CDK4 are also markers for distinguishing between dedifferentiated LGOS and conventional high-grade OSs [4]. Although the prognosis of LGOS is excellent, with 5-year survival rates of 90% with complete resection, in cases of dedifferentiated LGOS, chemotherapy is administered, and the prognosis is similar to that of conventional OS [6]. Toki et al. compared dedifferentiated LGOS with conventional OS, and the 5-year overall survival rates in the dedifferentiated and conventional OS groups were 85.7% and 77.1%, respectively. Dedifferentiated LGOS had a poorer response to a standard chemotherapy regimen than conventional OS, while the clinical outcomes were not markedly different [13]. The prognosis of dedifferentiated LGOS is associated with the timing of synchronous or metachronous dedifferentiation, or the histological amount of dedifferentiated areas remains. Low-grade components are inherently resistant to chemotherapy [13]. Therefore, standard chemotherapy may not be effective for dedifferentiated LGOS. Although our patient did not receive chemotherapy because of her refusal, there was no recurrence or lung metastasis 3 years after the final surgery.
